# Crystal structures of γ-glutamylmethylamide synthetase provide insight into bacterial metabolism of oceanic monomethylamine

**DOI:** 10.1074/jbc.RA120.015952

**Published:** 2020-11-21

**Authors:** Ning Wang, Xiu-Lan Chen, Chao Gao, Ming Peng, Peng Wang, Na Zhang, Fuchuan Li, Gui-Peng Yang, Qing-Tao Shen, Shengying Li, Yin Chen, Yu-Zhong Zhang, Chun-Yang Li

**Affiliations:** 1 State Key Laboratory of Microbial Technology, Marine Biotechnology Research Center, Shandong University, Qingdao, China; 2 College of Marine Life Sciences, and Frontiers Science Center for Deep Ocean Multispheres and Earth System, Ocean University of China, Qingdao, China; 3 Laboratory for Marine Biology and Biotechnology, Pilot National Laboratory for Marine Science and Technology, Qingdao, China; 4 School of Life Science and Technology, iHuman Institute, ShanghaiTech University, Shanghai, China; 5 National Glycoengineering Research Center and Shandong Key Laboratory of Carbohydrate Chemistry and Glycobiology, Shandong University, Qingdao, China; 6 Frontiers Science Center for Deep Ocean Multispheres and Earth System, Key Laboratory of Marine Chemistry Theory and Technology, Ministry of Education, Ocean University of China, Qingdao, China; 7 School of Life Sciences, University of Warwick, Coventry, United Kingdom

**Keywords:** γ-glutamylmethylamide synthetase (GmaS), crystal structure, enzyme mechanism, monomethylamine (MMA) metabolism, enzyme catalysis, protein complex, bacterial metabolism, AMPPCP, phosphomethylphosphonic acid-adenylate ester, AMPPNP, phosphoaminophosphonic acid-adenylate ester, GMA, γ-glutamylmethylamide, GmaS, γ-glutamylmethylamide synthetase, GS, glutamine synthetase, MetSox, methionine sulfoximine, MetSox-P, methionine sulfoximine phosphate, MMA, monomethylamine, MRC, marine Roseobacter clade, NMG, N-methylglutamate, PDB, protein data bank, *Rh*GmaS, GmaS from *Rhodovulum* sp. 12E13

## Abstract

Monomethylamine (MMA) is an important climate-active oceanic trace gas and ubiquitous in the oceans. γ-Glutamylmethylamide synthetase (GmaS) catalyzes the conversion of MMA to γ-glutamylmethylamide, the first step in MMA metabolism in many marine bacteria. The *gmaS* gene occurs in ∼23% of microbial genomes in the surface ocean and is a validated biomarker to detect MMA-utilizing bacteria. However, the catalytic mechanism of GmaS has not been studied because of the lack of structural information. Here, the GmaS from *Rhodovulum* sp. 12E13 (*Rh*GmaS) was characterized, and the crystal structures of apo-*Rh*GmaS and *Rh*GmaS with different ligands in five states were solved. Based on structural and biochemical analyses, the catalytic mechanism of *Rh*GmaS was explained. ATP is first bound in *Rh*GmaS, leading to a conformational change of a flexible loop (Lys287-Ile305), which is essential for the subsequent binding of glutamate. During the catalysis of *Rh*GmaS, the residue Arg312 participates in polarizing the γ-phosphate of ATP and in stabilizing the γ-glutamyl phosphate intermediate; Asp177 is responsible for the deprotonation of MMA, assisting the attack of MMA on γ-glutamyl phosphate to produce a tetrahedral intermediate; and Glu186 acts as a catalytic base to abstract a proton from the tetrahedral intermediate to finally generate glutamylmethylamide. Sequence analysis suggested that the catalytic mechanism of *Rh*GmaS proposed in this study has universal significance in bacteria containing GmaS. Our results provide novel insights into MMA metabolism, contributing to a better understanding of MMA catabolism in global carbon and nitrogen cycles.

Methylated amines, such as monomethylamine (MMA), dimethylamine, trimethylamine, and trimethylamine *N*-oxide, are widely distributed in marine environments and play important roles in marine carbon (C) and nitrogen (N) cycles (1, 2, 3). In the ocean, the major source of methylated amines is likely the breakdown of quaternary amine osmolytes, such as betaine, choline, and carnitine (4, 5). MMA, an ammonium analog, is an important component of marine one-carbon (C1) compounds (*i.e.*, compounds with no carbon–carbon bond) (6, 7); MMA provides carbon and energy sources as well as a nitrogen source for many microorganisms (2, 8, 9). In addition, the volatile MMA can enter the atmosphere from the oceans and has impacts on the global climate through participation of the formation of marine aerosols (3, 10).

There are two pathways identified from bacteria for aerobic MMA metabolism, a direct MMA-oxidation pathway and an indirect MMA-oxidation pathway (6). The direct MMA-oxidation pathway is only found in methylotrophic bacteria, through which MMA is metabolized to formaldehyde and ammonium by a single enzyme (4, 6). This enzyme is an MMA oxidase in gram-positive bacteria such as *Arthrobacter* (11) or an MMA dehydrogenase in gram-negative bacteria such as *Methylobacterium extorquens* and *Paracoccus denitrificans* (12, 13). In the indirect MMA-oxidation pathway, MMA is converted by γ-glutamylmethylamide synthetase (GmaS) to γ-glutamylmethylamide (GMA), which is further converted to *N*-methylglutamate (NMG) by NMG synthase, and finally to 5,10-methylenetetrahydrofolate (CH_2_ = THF) by NMG dehydrogenase (4, 8). Because NMG is an essential intermediate of the indirect MMA-oxidation pathway, this pathway is also termed as the NMG pathway.

Unlike the direct MMA-oxidation pathway which is only found in methylotrophic bacteria to date, the NMG pathway is adopted by both methylotrophic and nonmethylotrophic bacteria, in particular, by many bacteria of the marine *Roseobacter* clade (MRC) (14). MRC bacteria are ubiquitous and numerically abundant in marine environments (15, 16) and are important participants in MMA metabolism (14, 17, 18). It is estimated that half of the genomes of MRC strains contain *gmaS*, and this gene has been chosen as a biomarker to detect MMA-utilizing bacteria in the environment (6, 18). The *gmaS* gene occurs in ∼23% of microbial genomes in the surface ocean (17), suggesting that GmaS plays an important role in marine N and C cycles. However, despite that the biochemical characteristics of GmaS from several strains have been reported (19, 20, 21, 22), little is known about the molecular mechanism of GmaS catalyzing the conversion of MMA to GMA.

Sequence analysis indicates that GmaS is closely related to, but distinct from, the glutamine synthetase (GS) family, one of the oldest and most ubiquitously existing families of enzymes in biota (4, 23, 24). GmaS is more specific to MMA, whereas the natural substrate of GS enzymes is ammonia (19, 20, 25). This is consistent with the results of sequence analysis, showing that GmaS lacks key ammonia-binding residues that are conserved in GS enzymes (4, 21, 24, 26). GS enzymes can be divided into three distinct types, GSI, GSII, and GSIII (27, 28), and the crystal structures of GS have been determined (29, 30, 31). However, no crystal structure of GmaS is available to date, and the structural basis of GmaS to convert MMA to GMA remains obscure.

In this study, we report the structure of a GmaS enzyme and its molecular mechanism for the conversion of MMA to GMA. The *gmaS* gene from strain *Rhodovulum* sp. 12E13 (Rh*gmaS*) was expressed, and the recombinant *Rh*GmaS was purified and characterized. Six crystal structures of *Rh*GmaS in different states were solved. Based on structural and mutational assays, we proposed the molecular mechanism of *Rh*GmaS for the conversion of MMA to GMA. The results provide novel insights into MMA metabolism, leading to a better understanding of MMA catabolism in global C and N cycles.

## Results and discussion

### Characterization of *Rh*GmaS

**Figure 1:**
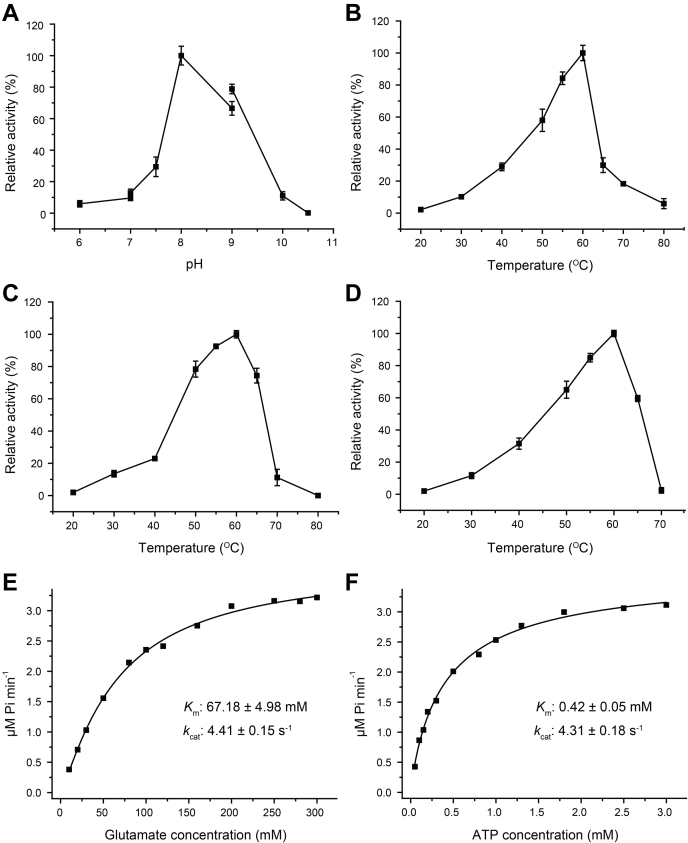
**Characterization of recombinant GmaS. The error bar represents the SD of triplicate experiments.***A*, the effect of pH on the enzymatic activity of *Rh*GmaS. *B*, the effect of temperature on the enzymatic activity of *Rh*GmaS. *C*, the effect of temperature on the enzymatic activity of GmaS from *R*. *pomeroyi* DSS-3. *D*, the effect of temperature on the enzymatic activity of GmaS from *Dinoroseobacter shibae* DFL12. *E*, Kinetic parameters of *Rh*GmaS for glutamate. *F*, kinetic parameters of *Rh*GmaS for ATP. GmaS, γ-glutamylmethylamide synthetase; *Rh*GmaS, GmaS from *Rhodovulum* sp. 12E13.

Full-length Rh*gmaS* of *R.* sp. 12E13 contains 1293 nucleotides and encodes a 430-amino-acid polypeptide, which shows 61% identity to the functional GmaS from a type strain of MRC, *Ruegeria pomeroyi* DSS-3 (18, 32). We chemically synthesized Rh*gmaS*, expressed it in *Escherichia coli* BL21 (DE3), and characterized the recombinant *Rh*GmaS. The recombinant *Rh*GmaS was active to catalyze the ligation of MMA and glutamate to produce GMA, with ATP and Mg^2+^ as cofactors. The optimal pH for *Rh*GmaS enzymatic activity was ∼8.0 (Fig. 1*A*), similar to that of *Methylovorus mays* No.9 GmaS (7.5–8.0) (21). The optimal temperature of *Rh*GmaS was 60 °C (Fig. 1*B*), which is higher than that of *Methylophaga* sp. AA-30 GmaS (40 °C) (19) and of *M. mays* No.9 GmaS (50 °C) (22). We noticed that the optimal temperatures of these GmaS enzymes are much higher than those of typical marine surface water column. Then, we purified two other GmaS homologs from MRC strains *R. pomeroyi* DSS-3 and *Dinoroseobacter shibae* DFL12. These two GmaS homologs also presented a high optimal temperature of 60 °C (Fig. 1, *C*–*D*), indicating that the relatively high optimal temperature is a common trait of GmaS proteins. Even so, *Rh*GmaS still maintains a specific activity of ∼0.71 μmol min^−1^ mg^−1^ at 20 °C, suggesting that *Rh*GmaS could work properly under the physiological temperature. The *K*_m_ of *Rh*GmaS for glutamate was 67.18 mM (Fig. 1*E*), and that for ATP was 0.42 mM (Fig. 1*F*). *Rh*GmaS exhibited a *K*_m_ value of 26.94 μM for MMA (Table 1), which is threefold lower than that of *M.* sp. AA-30 GmaS (89 μM) (19) and sixfold lower than that of *M. mays* No.9 GmaS (180 μM) (20). The micromolar level of *K*_m_ values of GmaS enzymes for MMA indicates that GmaS enzymes possess high affinities to MMA.

**Table 1:** Kinetic parameters for recombinant *Rh*GmaS with different substrates^a^

Substrate	*K*_m_ (μM)	*V*_max_ (μM min^−1^)	*k*_cat_ (s^−1^)
MMA	26.94 ± 1.73	3.58 ± 0.11	4.18 ± 0.13
Ethylamine	58.10 ± 4.49	3.51 ± 0.11	4.09 ± 0.13
Hydroxylamine	211.03 ± 9.87	3.56 ± 0.05	4.15 ± 0.06
Propylamine	1.79 × 10^3^ ± 0.12 × 10^3^	3.63 ± 0.08	4.23 ± 0.09
NH_4_Cl	8.72 × 10^3^ ± 0.97 × 10^3^	3.65 ± 0.17	4.26 ± 0.20
DMA	16.07 × 10^3^ ± 1.15 × 10^3^	2.20 ± 0.09	2.57 ± 0.10
TMA	22.91 × 10^3^ ± 1.50 × 10^3^	2.13 ± 0.06	2.48 ± 0.07

DMA, dimethylamine; MMA, monomethylamine; TMA, trimethylamine.

a The experiments were performed at pH 8.0, 30 °C. The data shown in the table are from triplicate experiments (means ± SDs).

Amine donors, such as hydroxylamine and ethylamine, can replace ammonia as substrate for some GS enzymes (20, 24, 30). To determine whether *Rh*GmaS can catalyze different ammonia analogs, we analyzed the substrate specificity of *Rh*GmaS (Table 1). In addition to MMA, *Rh*GmaS can accept ethylamine, hydroxylamine, propylamine, ammonium chloride, dimethylamine, or trimethylamine as a substrate, indicating that this enzyme has a relatively broad substrate specificity. The *K*_m_ of *Rh*GmaS for MMA was the lowest among the tested ammonia analogs, while the *K*_m_ for NH_4_Cl is much higher (Table 1), suggesting that MMA is likely the natural substrate of *Rh*GmaS. This result is consistent with the previous reports that GmaS prefers MMA as its substrate rather than ammonia (4, 19, 20, 24). To investigate whether the N-terminal His-tag would affect the kinetic properties of the recombinant *Rh*GmaS, we used thrombin to cut off the His-tag and measured the kinetic parameters of *Rh*GmaS without His-tag (Fig. S1), which were similar to *Rh*GmaS with His-tag. This result indicates that the presence of the His-tag has little effect on the kinetic properties of the enzyme. The *Rh*GmaS proteins used in this study all contained the His-tag, unless otherwise noted.

### Overall structure of *Rh*GmaS

**Figure 2:**
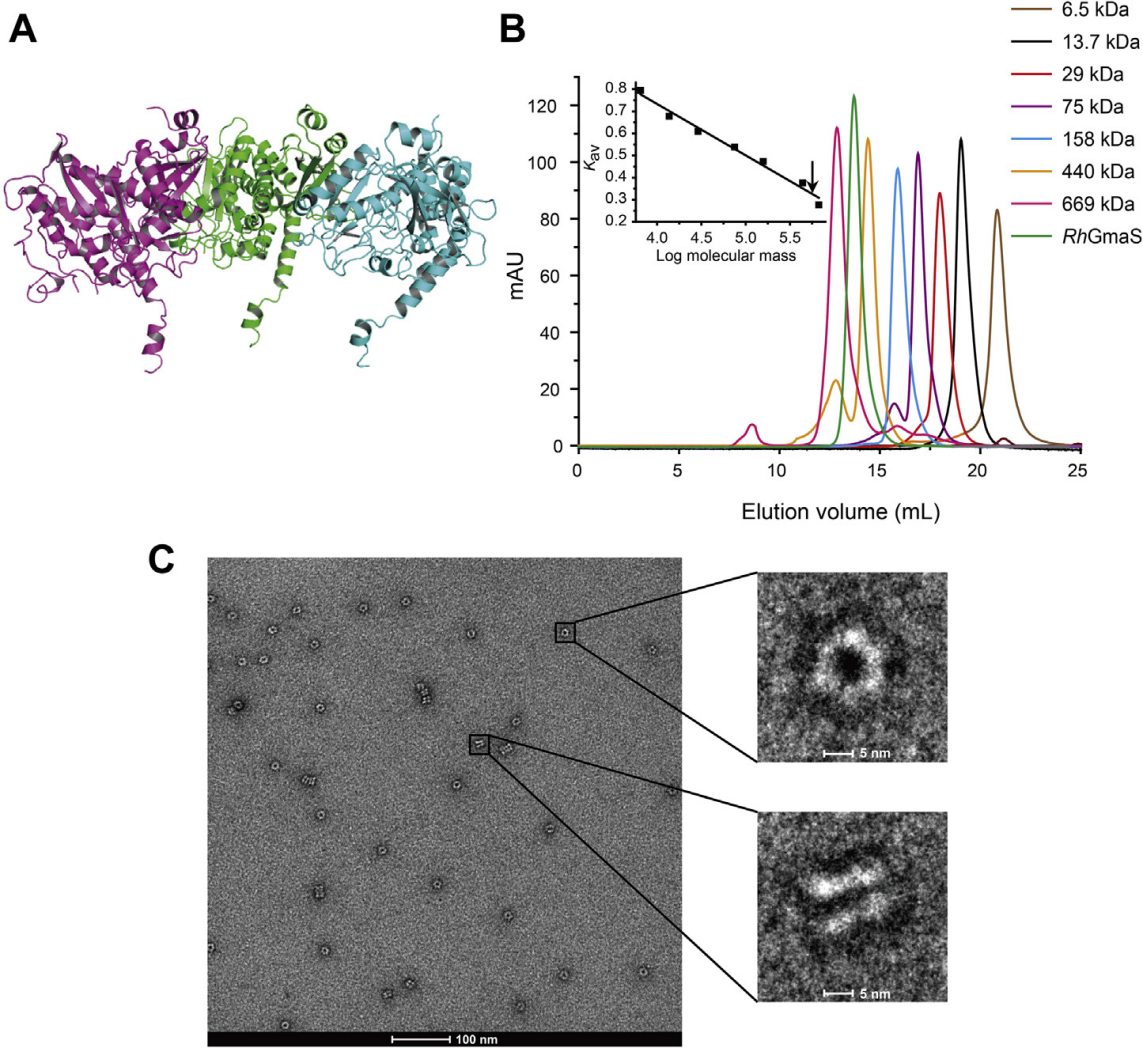
**Overall structural analysis of *Rh*GmaS.***A*, the overall structure of *Rh*GmaS. There are three *Rh*GmaS monomers arranged as a trimer in an asymmetric unit. The three monomers are colored in *purple*, *green*, and *blue*, respectively. *B*, gel filtration analysis of *Rh*GmaS. Inset, semilog plot of the molecular mass of all standards used versus their *K*_av_ values. The *black arrow* indicates the position of *Rh*GmaS *K*_av_ value interpolated in the regression line. The predicted molecular mass of *Rh*GmaS monomer is 46.64 kDa. *C*, the negative-staining electron micrograph of *Rh*GmaS. *Rh*GmaS consists of two hexameric rings, with each ring containing six monomers. *Rh*GmaS, GmaS from *Rhodovulum* sp. 12E13.

To gain insight into the catalytic mechanism of *Rh*GmaS, the crystal structure of apo-*Rh*GmaS was solved (Table 2). There are three monomers arranged as a trimer in an asymmetric unit (Fig. 2*A*), with each monomer composed of 15 α-helices and 13 β-strands. However, gel filtration analysis demonstrated that *Rh*GmaS is a dodecamer in solution (Fig. 2*B*), which is consistent with the result of electron microscopic analysis (Fig. 2*C*). The negative staining electron micrograph clearly showed that *Rh*GmaS consists of two hexameric rings, with each ring containing six monomers (Fig. 2*C*). Thus, *Rh*GmaS should function as a dodecamer in the solution. During structural refinement, we could not find the electron densities of the N-terminal His-tag, suggesting that this tag is flexible. The electron microscopic analysis showed that *Rh*GmaS without His-tag still maintains a dodecamer containing two hexameric rings in the solution (Fig. S2). These data suggest that the presence of the His-tag has little effect on the structural properties of *Rh*GmaS. The overall structure of *Rh*GmaS is similar to the structure of a GS enzyme from *Bacillus subtilis* (protein data bank [PDB] code: 4LNI), with an RMSD of 0.86 Å between these two structures. The *B. subtilis* GS also functions as a dodecamer (33).

**Table 2:** Crystallographic data collection and refinement of *Rh*GmaS

Parameters	Apo-*Rh*GmaS	*Rh*GmaS–AMPPCP	*Rh*GmaS–AMPPNP–MetSox	*Rh*GmaS–ADP–MetSox-P	*Rh*GmaS–ADP-C1	*Rh*GmaS–ADP-C2
Diffraction data						
Space group	I222	I222	I222	I222	I222	I222
Unit cell						
a, b, c (Å)	115.9, 179.0, 192.2	110.9, 176.7, 191.8	115.6, 174.5, 190.1	116.6, 174.7, 190.6	114.7, 176.0, 190.5	113.4, 177.6, 190.5
α, β, γ (°)	90.0, 90.0, 90.0	90.0, 90.0, 90.0	90.0, 90.0, 90.0	90.0, 90.0, 90.0	90.0, 90.0, 90.0	90.0, 90.0, 90.0
Resolution range (Å)	50.0–2.8 (2.90–2.80) a	50.0–1.96 (1.99–1.96)	50.0–2.3 (2.34–2.30)	50.0–2.1 (2.18–2.10)	50.0–2.3 (2.34–2.30)	50.0–2.3 (2.38–2.30)
Redundancy	11.3 (10.7)	4.6 (2.9)	6.4 (6.0)	4.0 (3.0)	11.5 (10.5)	13.4 (12.6)
Completeness (%)	98.6 (99.1)	98.1 (87.7)	100.0 (100.0)	97.5 (94.5)	99.5 (100.0)	100.0 (100.0)
*R*_merge_^b^	0.1 (0.6)	0.1 (0.4)	0.1 (0.4)	0.1 (0.5)	0.2 (0.4)	0.1 (0.5)
*I*/σ*I*	54.0 (14.7)	14.2 (1.8)	18.6 (2.9)	21.8 (3.3)	34.9 (6.4)	22.6 (5.4)
Refinement statistics						
*R*_work_/*R*_free_	0.20/0.25	0.17/0.20	0.16/0.20	0.16/0.20	0.17/0.22	0.18/0.21
RMSD from ideal geometry						
Bond lengths (Å)	0.009	0.007	0.007	0.007	0.007	0.007
Bond angles (°)	1.2	1.1	1.1	1.1	1.1	1.1
Ramachandran plot (%)						
Favored	93.6	97.2	97.1	97.6	95.7	96.3
Allowed	6.2	2.8	2.9	2.3	4.1	3.7
Outliers	0.2	0	0	0.1	0.2	0
Overall B-factors (Å^2^)	57.1	18.9	37.2	24.3	35.7	27.1

a Numbers in parentheses refer to data in the highest resolution shell.

b *R*_merge_=∑_*hkl*_∑_*i*_|*I*(*hkl*)_*i*_ -<*I*(*hkl*)>|/∑_*hkl*_∑_*i*_*I*(*hkl*)_*i*_, where *I* is the observed intensity, and *I*(*hkl*)_*i*_ represents the observed intensity of each unique reflection.

We further cocrystallized *Rh*GmaS with ATP/ADP, glutamate, and MMA. However, after we solved the structures, we could not find glutamate or MMA bound in *Rh*GmaS, and ATP was hydrolyzed to ADP during crystallization. We then tried to cocrystallize *Rh*GmaS with substrate analogs. Phosphoaminophosphonic acid-adenylate ester (AMPPNP) and phosphomethylphosphonic acid-adenylate ester (AMPPCP) are nonhydrolyzable ATP analogs. Methionine sulfoximine (MetSox) is an analog of glutamate, with a methylsulfoximine group replacing the carboxyl group of glutamate. MetSox can be phosphorylated to form a transition analog, methionine sulfoximine phosphate (MetSox-P) (33). Finally, the crystal structures of the *Rh*GmaS–AMPPCP complex, *Rh*GmaS–AMPPNP–MetSox complex, *Rh*GmaS–ADP–MetSox-P complex, and *Rh*GmaS–ADP complexes in two conformations (*Rh*GmaS–ADP-C1 and *Rh*GmaS–ADP-C2) were solved (Table 2), which provide us abundant structural information for further mechanistic analysis. All the solved *Rh*GmaS structures contain three molecules arranged as a trimer in an asymmetric unit, suggesting that the dodecameric *Rh*GmaS has a relatively high symmetry.

### Residues involved in the binding of ATP, glutamate and Mg^2+^

**Figure 3:**
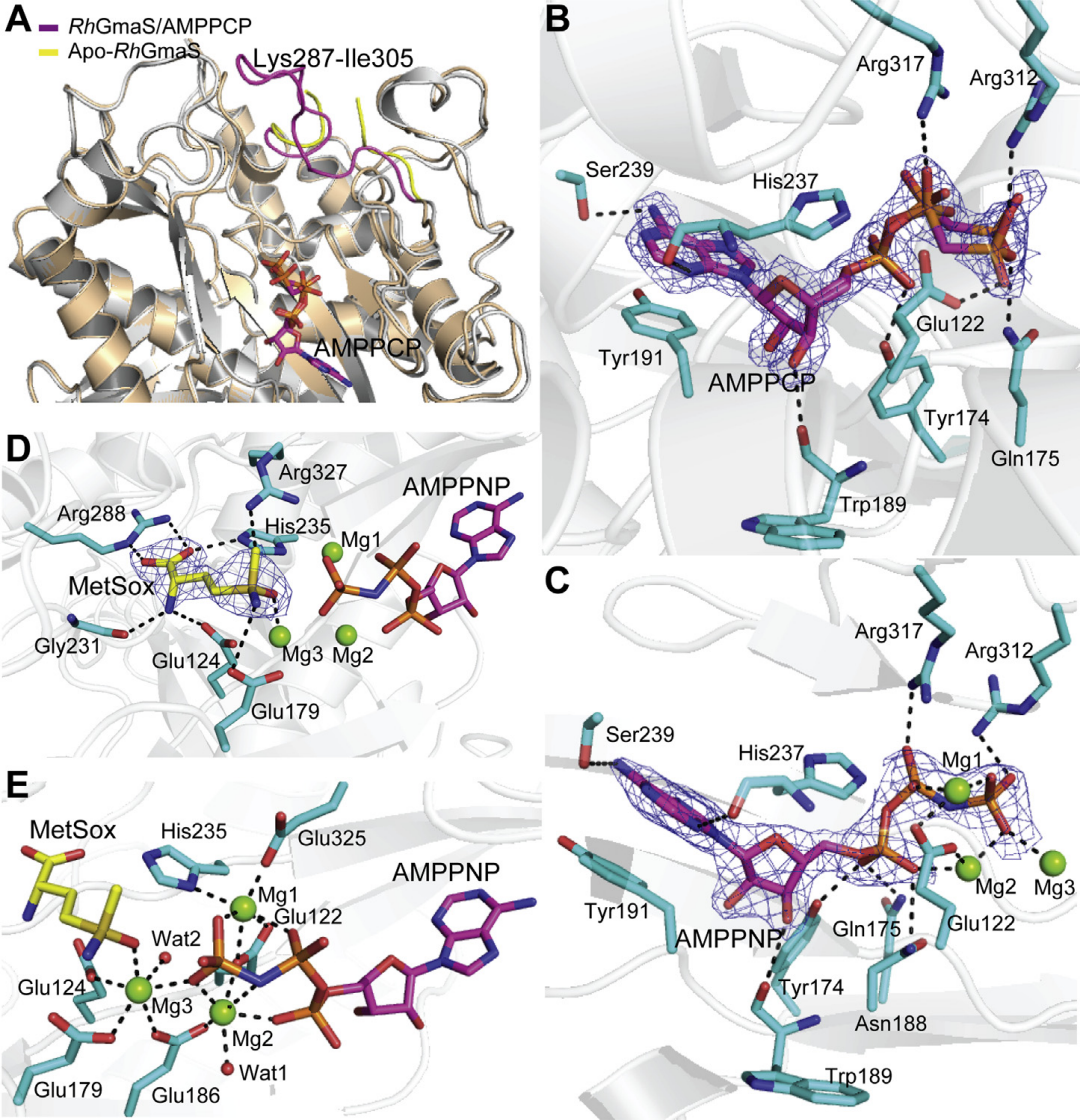
**Structural analysis of *Rh*GmaS–AMPPCP and *Rh*GmaS–AMPPNP–MetSox complexes.***A*, structural alignment of *Rh*GmaS–AMPPCP and apo-*Rh*GmaS. The loop regions (Lys287-Ile305) of *Rh*GmaS–AMPPCP (*magenta*) and that of apo-*Rh*GmaS (*yellow*) are highlighted. *B*, residues involved in binding AMPPCP. AMPPCP is colored in *magenta*, and *Rh*GmaS residues are colored in *cyan*. The 2*F*_*o*_ - *F*_*c*_ densities for AMPPCP are contoured in *blue* at 1.5σ. The possible hydrogen bonds are represented by *dashed lines*. *C*, residues and atoms involved in binding AMPPNP. AMPPNP is colored in *magenta*, and *Rh*GmaS residues are colored in *cyan*. The 2*F*_*o*_ - *F*_*c*_ densities for AMPPNP are contoured in *blue* at 2.0σ. *D*, *Rh*GmaS residues involved in binding MetSox. MetSox is colored in *yellow*. The 2*F*_*o*_ - *F*_*c*_ densities for MetSox are contoured in *blue* at 2.0σ. *E*, residues and ligands involved in binding Mg^2+^. The possible hydrogen bonds are represented by *dashed lines*. GmaS, γ-glutamylmethylamide synthetase; *Rh*GmaS, GmaS from *Rhodovulum* sp. 12E13; MetSox, methionine sulfoximine.

In the crystal structure of apo-*Rh*GmaS, the electron densities of residues Pro292-Trp300 are rather poor, and we could not place these residues during structural refinement, indicating a relatively high flexibility of this region. However, in the crystal structure of the *Rh*GmaS–AMPPCP complex, the electron densities of this region are clear, and the conformation of the loop Lys287-Ile305 is different from that in the apo-*Rh*GmaS structure (Fig. 3*A*), suggesting that the binding of the ATP molecule will lead to the conformation change of the loop Lys287-Ile305.

Residues involved in binding ATP are identified based on the crystal structure of the *Rh*GmaS–AMPPCP complex. The residue Tyr191 forms pi–pi stacking interaction with the adenine moiety of ATP, and residues His237 and Ser239 interact with the adenine moiety through hydrogen bonds (Fig. 3*B*). Trp189 forms a hydrogen bond with the ribose moiety, and residues Glu122, Tyr174, Gln175, Arg312, and Arg317 participate in binding the phosphate moieties of ATP (Fig. 3*B*). We noticed that the AMPPCP molecule exhibits two conformations in the *Rh*GmaS–AMPPCP complex (Fig. 3*B*), suggesting that the binding of ATP without glutamate and Mg^2+^ may not be tight.

Despite many attempts, we did not obtain the crystal structure of the *Rh*GmaS–glutamate complex, probably due to the low affinity of *Rh*GmaS for glutamate (Fig. 1*E*). Instead, we solved the crystal structure of the *Rh*GmaS–AMPPNP–MetSox complex (Table 2), which can reflect the binding mode of ATP and glutamate. The overall structure of the *Rh*GmaS–AMPPNP–MetSox complex is similar to that of the *Rh*GmaS–AMPPCP complex, with an RMSD of 0.19 Å between these two structures. Three Mg^2+^ were also identified in the crystal structure of the *Rh*GmaS–AMPPNP–MetSox complex. The locations of AMPPNP, MetSox, and three Mg^2+^ are well defined based on the electron density map. All three Mg^2+^ participate in binding AMPPNP–ATP, which partially changes the binding mode of ATP. Compared with the *Rh*GmaS–AMPPCP complex, the residue Glu122 does not interact with ATP directly but through Mg1 and Mg2, and Asn188 forms a new hydrogen bond with ATP (Fig. 3*C*).

The binding of MetSox in *Rh*GmaS mainly depends on hydrogen bonds between its hydrophilic atoms and *Rh*GmaS residues Glu124, Glu179, Gly231, His235, Arg288, and Arg327 (Fig. 3*D*). Interestingly, Arg288 is located in the flexible loop Lys287-Ile305, and the conformations of this loop in the *Rh*GmaS–AMPPCP complex and the *Rh*GmaS–AMPPNP–MetSox complex are identical. This result indicates that the conformational change of loop Lys287-Ile305 due to ATP binding will facilitate the subsequent binding of glutamate.

Three Mg^2+^ are coordinated by MetSox, AMPPNP, two water molecules, and *Rh*GmaS residues Glu122, Glu124, Glu179, Glu186, His235, and Glu325 (Fig. 3*E*). In fact, when we cocrystallized *Rh*GmaS and AMPPCP, we also added Mg^2+^ in the protein solution, but the fact that no Mg^2+^ presents in the *Rh*GmaS–AMPPCP complex suggests that Mg^2+^ is bound after the binding of glutamate.

**Figure 4:**
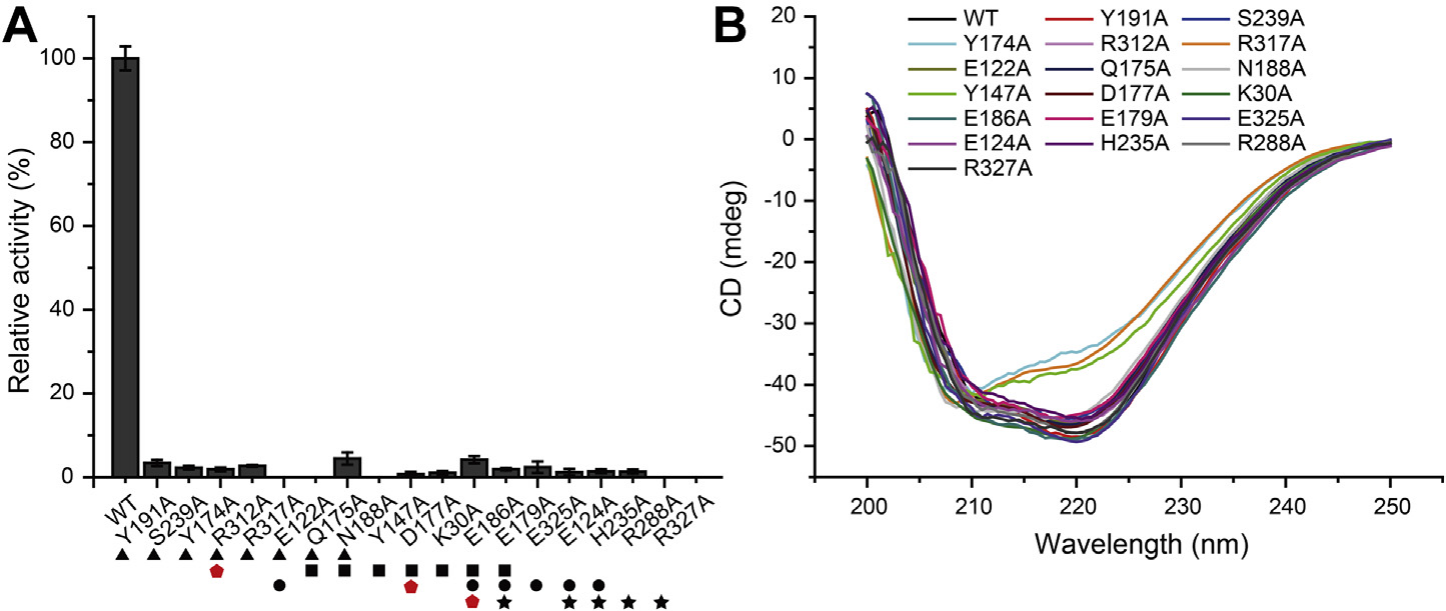
**Mutational analysis of important residues of *Rh*GmaS that may participate in binding substrate and catalysis.***A*, the enzymatic activities of WT *Rh*GmaS and its mutants. The activity of WT *Rh*GmaS is taken as 100%. Residues involved in binding ATP, MMA, Mg^2+^, and glutamate are marked with *black triangles*, *squares*, *dots*, and *stars*, respectively. *Red pentagons* indicate the catalytic residues of *Rh*GmaS. The error bar represents SD of triplicate experiments. *B*, CD spectra of WT *Rh*GmaS and its mutants. MMA, monomethylamine; *Rh*GmaS, GmaS from *Rhodovulum* sp. 12E13; CD, circular-dichroism.

To confirm the importance of *Rh*GmaS residues involved in binding ATP, glutamate, and Mg^2+^, we generated site-directed mutations to these residues and quantified the enzymatic activities of the mutants. All the mutations dramatically decreased the activity of *Rh*GmaS (Fig. 4*A*), suggesting that these residues play important roles during the catalysis of *Rh*GmaS. Circular-dichroism (CD) spectroscopy analysis showed that the secondary structures of the mutants exhibited little deviation from that of WT *Rh*GmaS with the exceptions of Tyr174Ala and Arg317Ala (Fig. 4*B*), indicating that the decreases in the enzymatic activities of most mutants are caused by residue replacement rather than structural changes. Mutation of Tyr174 or Arg317 to Ala led to a change of the secondary structure of *Rh*GmaS (Fig. 4*B*), which may be caused by the differences of side-chain properties between Tyr or Arg and Ala.

### The catalytic residues of *Rh*GmaS

**Figure 5:**
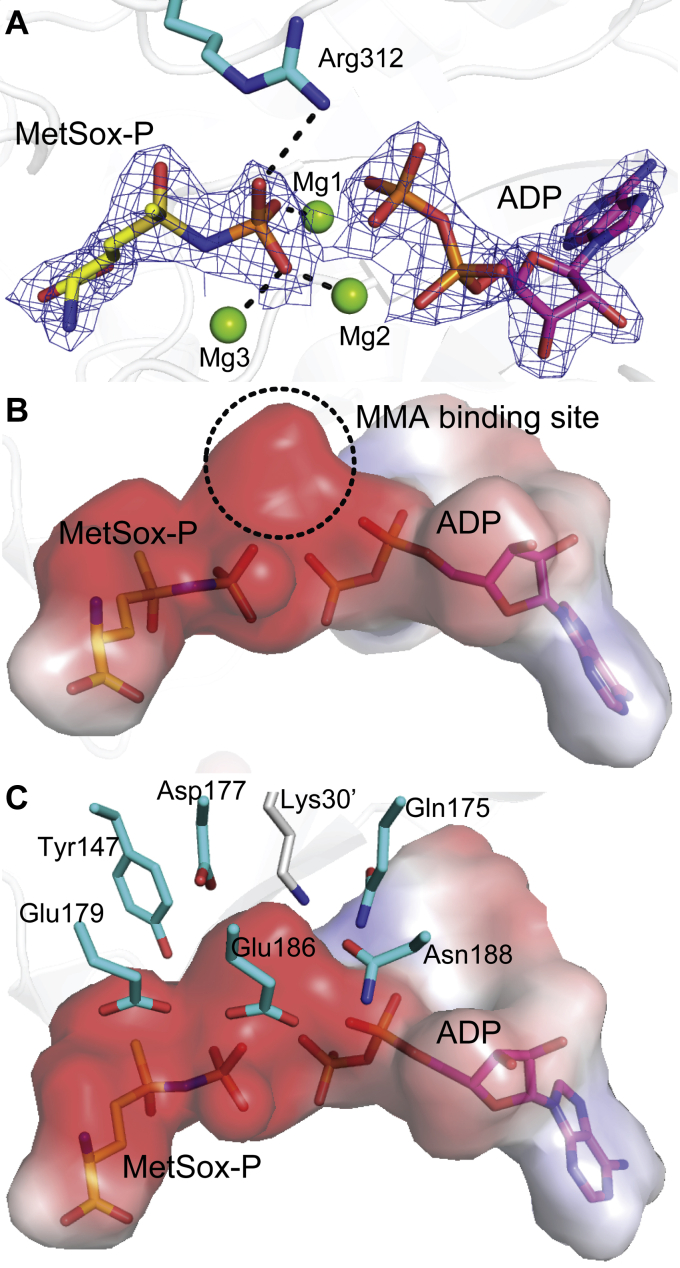
**Structural analysis of the *Rh*GmaS–ADP–MetSox-P complex.***A*, the positions of MetSox-P and ADP. The 2*F*_*o*_ - *F*_*c*_ densities for MetSox-P and ADP are contoured in *blue* at 2.0σ. *B*, the MMA binding site of *Rh*GmaS. *C*, *Rh*GmaS residues composing the negative-charged MMA binding site. MMA, monomethylamine; *Rh*GmaS, GmaS from *Rhodovulum* sp. 12E13; MetSox, methionine sulfoximine.

Based on the catalytic mechanism of GS enzymes (33), it is likely that *Rh*GmaS catalyzes a two-step reaction: in the first step, glutamate is phosphorylated, leading to the formation of γ-glutamyl phosphate, which is subsequently attacked by MMA to yield GMA in the second step. To obtain a transition state of *Rh*GmaS catalysis, we cocrystallized *Rh*GmaS with ATP, MgCl_2_, and MetSox and obtained the crystal structure of the *Rh*GmaS–ADP–MetSox-P complex (Table 2). In this structure, Arg312 and Mg^2+^ interact with the phosphate group of MetSox-P (Fig. 5*A*), indicating that Arg312 and Mg^2+^ are involved in stabilizing the transition state.

To obtain the crystal structure of *Rh*GmaS in complex with MMA, we tried cocrystallization and soaking methods, but all attempts failed. However, structure analysis of the *Rh*GmaS–ADP–MetSox-P (the γ-glutamyl phosphate mimic) complex provided us some clues of the MMA binding site. A negative-charged pocket close to the ADP and MetSox-P molecules is observed in the complex (Fig. 5*B*), which is mainly formed by hydrophilic residues, including Tyr147, Gln175, Asp177, Glu179, Glu186, Asn188, and Lys30' (where a prime indicates the neighboring subunit) (Fig. 5*C*). Because MMA is alkaline, it is likely that *Rh*GmaS attracts MMA through electrostatic interactions. After MMA enters the active site, it will be deprotonated before its attack on the γ-glutamyl phosphate of ATP. Structure analysis suggests that Asp177 or Glu186 is the potential residue to deprotonate MMA (Fig. 5*C*). Previous studies on GS enzymes indicate that the attack of ammonia on γ-glutamyl phosphate will generate a tetrahedral intermediate, which is further deprotonated before the final formation of glutamine (33, 34). In the case of *Rh*GmaS, the attack of MMA on γ-glutamyl phosphate may generate a similar tetrahedral intermediate. Because Glu186 is closer to the phosphoryl group of MetSox-P than Asp177 in the *Rh*GmaS–ADP–MetSox-P complex, we deduced that Glu186 deprotonates the tetrahedral intermediate while Asp177 deprotonates MMA during *Rh*GmaS catalysis.

Mutations of residues composing the MMA binding site and directly participating in the catalysis decreased the activity of *Rh*GmaS significantly (Fig. 4*A*), suggesting a crucial role of these residues. CD spectroscopy analysis suggested that the decreases in the enzymatic activities of most mutants resulted from residue replacements, while the mutation Tyr147Ala changed the secondary structure of *Rh*GmaS (Fig. 4*B*).

### Conformational change of *Rh*GmaS after catalysis

**Figure 6:**
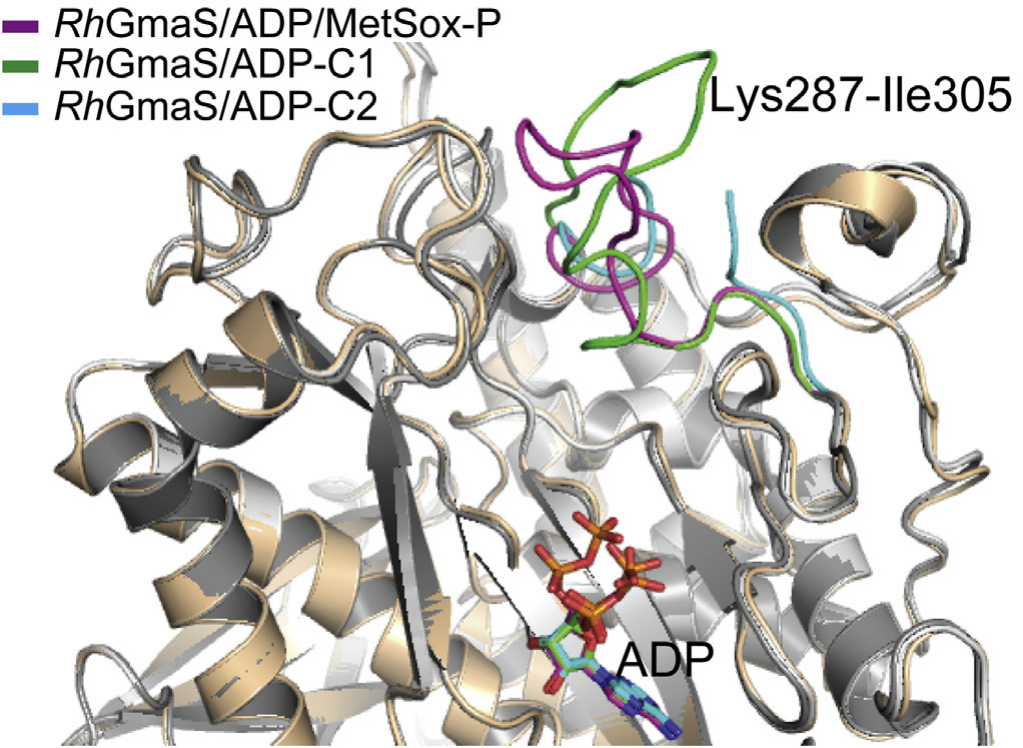
**Analysis of the conformational change of *Rh*GmaS after catalysis.** The loop regions (Lys287-Ile305) of *Rh*GmaS–ADP–MetSox-P (*magenta*), of *Rh*GmaS–ADP-C1 (*green*), and of *Rh*GmaS–ADP-C2 (*blue*) are highlighted. ADP molecules are shown as *sticks*. *Rh*GmaS, GmaS from *Rhodovulum* sp. 12E13; MetSox, methionine sulfoximine.

After GMA is generated, GMA, ATP, and Mg^2+^ will be released from the catalytic pocket of *Rh*GmaS, enabling *Rh*GmaS to be ready for the next catalytic cycle. We determined the crystal structures of two *Rh*GmaS–ADP complexes in different conformations (*Rh*GmaS–ADP-C1 and *Rh*GmaS–ADP-C2), which may reflect the events after catalysis. When structures of *Rh*GmaS–ADP–MetSox-P, *Rh*GmaS–ADP-C1, and *Rh*GmaS–ADP-C2 are aligned (Fig. 6), it can be seen that the conformation of ADP in *Rh*GmaS–ADP–MetSox-P is different from those in *Rh*GmaS–ADP-C1 and *Rh*GmaS–ADP-C2, and the conformations of the loop region Lys287-Ile305 in these three structures are different. Because Arg288 in the loop region participates in binding glutamate (Fig. 3*D*), the conformational change of the loop region indicates that GMA and Mg^2+^ are released firstly after the catalysis. Without the binding of GMA and Mg^2+^, the binding of ADP becomes relatively loose and will be finally released from the active site of *Rh*GmaS.

### The catalytic cycle of *Rh*GmaS

**Figure 7:**
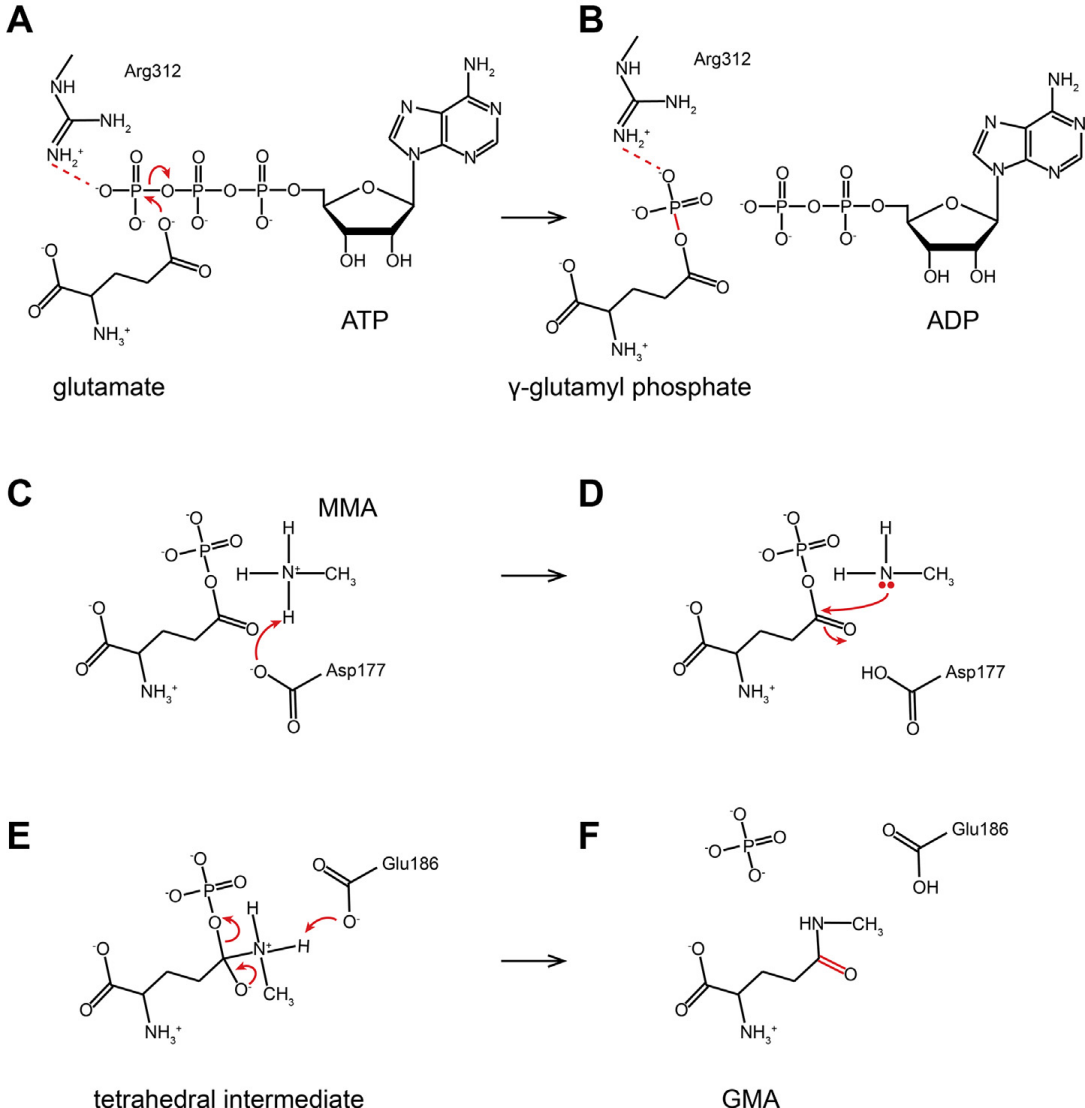
**The catalytic mechanism of *Rh*GmaS converting MMA to GMA.***A*, the residue Arg312 directly interacts with ATP γ-phosphate, which helps polarize the γ-phosphate and facilitates the nucleophilic attack of glutamate. *B*, Arg312 interacts with the phosphate group of γ-glutamyl phosphate to stabilize the transition state. *C*, MMA is deprotonated by the residue Asp177. *D*, MMA attacks γ-glutamyl phosphate to produce a tetrahedral intermediate. *E*, Glu186 abstracts a proton from the tetrahedral intermediate. *F*, GMA is generated. GMA, γ-glutamylmethylamide; MMA, monomethylamine; *Rh*GmaS, GmaS from *Rhodovulum* sp. 12E13.

Based on structural and biochemical assays, the catalytic cycle of *Rh*GmaS converting MMA to GMA is proposed (Fig. 7). In the first step of *Rh*GmaS catalysis, ATP is first bound in *Rh*GmaS, which leads to a conformational change of the loop Lys287-Ile305, assisting the subsequent binding of glutamate. The residue Arg312 and Mg^2+^ directly interact with ATP γ-phosphate (Fig. 3*C*), which helps polarize the γ-phosphate and facilitates the nucleophilic attack of glutamate (33) (Fig. 7*A*). After glutamate is phosphorylated, Arg312 forms a hydrogen bond with the phosphate group of γ-glutamyl phosphate to stabilize the transition state (Fig. 7*B*). In the second step, MMA is attracted by *Rh*GmaS and enters into the binding pocket. The residue Asp177 deprotonates MMA (Fig. 7*C*), enabling MMA to attack γ-glutamyl phosphate (Fig. 7*D*). A tetrahedral intermediate is then produced with the attack of MMA (Fig. 7*E*). Finally, Glu186 abstracts a proton from the tetrahedral intermediate, and GMA is generated (Fig. 7*F*). Then *Rh*GmaS releases GMA and ADP and is ready for the next catalytic cycle.

**Figure 8:**
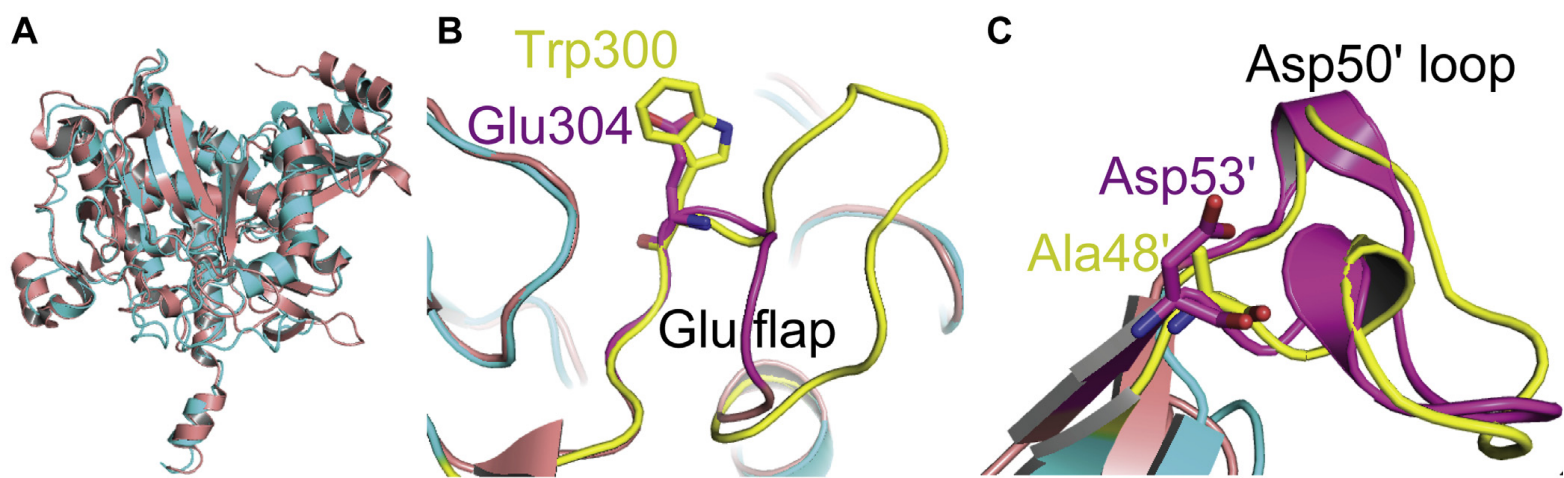
**Structural comparison between *Rh*GmaS and the GS enzyme from *Bacillus subtilis* (PDB code: 4LNI).***A*, superimposition of the *Rh*GmaS structure (*cyan*) onto the GS structure (*red*). *B*, the Glu flap of GS (*purple*) and the corresponding loop in *Rh*GmaS (*yellow*). The conserved glutamate residue (Glu304 in *B. subtilis* GS) and the corresponding residue in *Rh*GmaS (Trp300) are shown as *sticks*. *C*, the Asp50' loop of GS (*purple*) and the corresponding loop in *Rh*GmaS (*yellow*). The conserved aspartate residue (Asp53' in *B. subtilis* GS) and the corresponding residue in *Rh*GmaS (Ala48') are shown as *sticks*. GS, glutamine synthetase; PDB, protein data bank; *Rh*GmaS, GmaS from *Rhodovulum* sp. 12E13.

**Figure 9:**
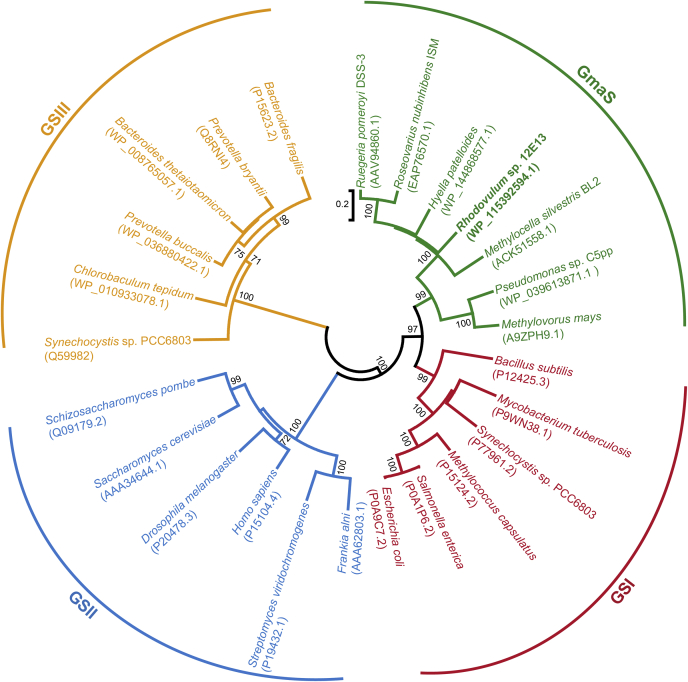
**The neighbor-joining phylogenetic tree of GSI, GSII, GSIII, and GmaS.** Phylogenetic analyses were carried out using the MEGA, version 6.0 (39). GmaS, γ-glutamylmethylamide synthetase.

Despite that the overall structure of *Rh*GmaS is similar to that of the GS enzyme from *B. subtilis* (PDB code: 4LNI) (33) (Fig. 8*A*), the catalytic processes of *Rh*GmaS and GS are different. Two loops (the Glu flap and the Asp50' loop) play key roles in the catalytic process of GS (25, 33). The Glu flap of GS possesses several functions: (i) guarding substrate entry and product release; (ii) shielding the γ-glutamyl phosphate from aberrant hydrolysis; and more importantly, (iii) harboring the conserved glutamate residue (Glu304 in *B. subtilis* GS), which acts as the catalytic base for the final attack on the tetrahedral intermediate (25, 33). Structural analysis suggests that the loop Lys287-Ile305 of *Rh*GmaS corresponds to the Glu flap in GS (Fig. 8*B*). However, there is no glutamate residue in this loop, and the corresponding residue of Glu304 in *B. subtilis* GS is Trp300 in *Rh*GmaS (Fig. 8*B*). For *Rh*GmaS, this loop region only acts as a gate for substrate entry and product release, and Glu186 is the catalytic residue to attack the tetrahedral intermediate. The aspartate residue in the Asp50' loop is strictly conserved in GS, which has two functions: (i) participating in binding ammonium and (ii) deprotonating the ammonium in the active site to create ammonia (25, 33). The corresponding region of the Asp50' loop in *Rh*GmaS is Phe47'-Ala61', and the corresponding residue of Asp50' in *Rh*GmaS is Ala48' (Fig. 8*C*). Structural analysis suggests that no residue of *Rh*GmaS in the region Phe47'-Ala61' participates in binding MMA, and Lys30' is the only residue from the neighboring subunit involved in composing the MMA binding site (Fig. 5*C*). The result of structural analysis confirmed the previous sequence analysis that GmaS lacks the key ammonia-binding residue (4, 21, 24, 26), highlighting the differences between GmaS and GS. Moreover, phylogenetic analysis indicated that GmaS proteins form a separate clade from GSI, GSII, and GSIII enzymes (Fig. 9), which is consistent with previous studies (4, 32), suggesting the divergent evolution of GmaS from GS.

### Universality of the catalytic mechanism of *Rh*GmaS

**Figure 10:**
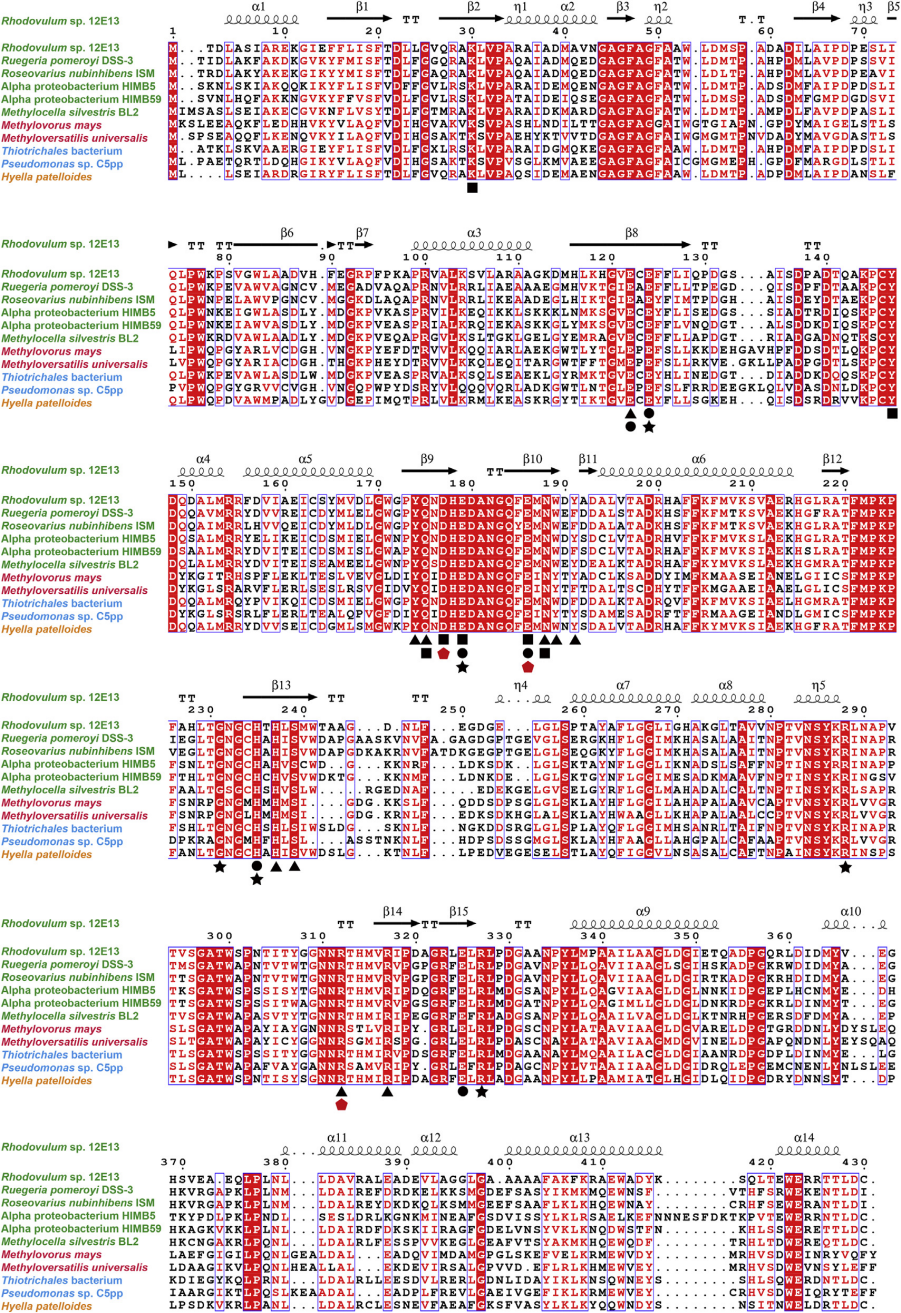
**Sequence alignment of GmaS proteins.** Residues involved in binding ATP, MMA, Mg^2+^, and glutamate are marked with *black triangles*, *squares*, *dots*, and *stars*, respectively. *Red pentagons* indicate the catalytic residues. GmaS proteins from strains of different classes are colored as follows: *green* for *Alphaproteobacteria*, *red* for *Betaproteobacteria*, *blue* for *Gammaproteobacteria*, and *yellow* for *Cyanophyceae*. GmaS, γ-glutamylmethylamide synthetase; MMA, monomethylamine.

GmaS homologs widely occur in marine bacteria, including MRC and SAR11 clade (17), supporting a significant role of MMA metabolism in marine C and N cycles. Moreover, many soil bacteria are also reported to possess GmaS homologs, such as *Methylocella silvestris* BL2 (4) and *Pseudomonas* sp. C5pp (24). To study the ubiquity of the *Rh*GmaS catalytic mechanism, we carried out sequence alignment of GmaS homologs from different strains (Fig. 10). The result showed that most of the residues involved in binding ATP, glutamate, MMA, and Mg^2+^ are highly conserved in GmaS homologs (Fig. 10). In particular, the catalytic residues Arg312, Asp177, and Glu186 are strictly conserved in all GmaS homologs (Fig. 10), suggesting that the molecular mechanism of *Rh*GmaS to convert MMA to GMA is likely adopted by most of other bacterial GmaSs.

## Conclusion

In this study, we solved the crystal structures of *Rh*GmaS in different states for the first time. Based on structural and mutational analysis, we explained the catalytic mechanism of *Rh*GmaS for the conversion of MMA to GMA in detail. Sequence alignment indicated that the proposed molecular mechanism of *Rh*GmaS has universal significance. Our results provide novel insights into marine MMA metabolism and broaden our understanding of the biogeochemical cycles of C and N.

## Experimental procedures

### Bacterial strains and growth conditions

The *E*. *coli* strains DH5α and BL21 (DE3) were grown in the lysogeny broth medium at 37 °C.

### Gene synthesis, point mutation, and protein expression and purification

The full-length Rh*gmaS* gene from *R.* sp. 12E13 was synthesized by the Beijing Genomics Institute (China). The gene was then cloned into the pET-28a vector containing an N-terminal His-tag (Novagen). All point mutations of *Rh*GmaS were created by the PCR-based method and confirmed by DNA sequencing. The *Rh*GmaS protein and all the mutants were expressed in *E*. *coli* BL21 (DE3). The recombinant strains were grown at 37 °C in the lysogeny broth medium containing 30 μg/ml kanamycin and then were induced by 0.5-mM IPTG at 18 °C for 16 h. All proteins were purified first by Ni^2+^ nitrilotriacetic acid resin (Qiagen, Germany) and then by anion-exchange chromatography on a Source 15Q column (GE Healthcare) with 0- to 1-M NaCl in 50-mM Tris-HCl (pH 8.0). The eluted proteins were further fractionated by gel filtration on a Superose 6 column (GE Healthcare) with the buffer containing 10-mM Tris HCl (pH 8.0) and 100-mM NaCl. To prepare *Rh*GmaS protein without His-tag, we digested the purified *Rh*GmaS by thrombin (Solarbio, China) at 4 °C overnight, and then the mixtures were fractionated by gel filtration on a Superose 6 column.

### Gel filtration analysis

The Superose 6 column was calibrated in the buffer containing 10-mM Tris HCl (pH 8.0) and 100-mM NaCl using the following standards from GE Healthcare: thyroglobulin (669 kDa), ferritin (440 kDa), aldolase (158 kDa), conalbumin (75 kDa), carbonic anhydrase (29 kDa), ribonuclease A (13.7 kDa), and aprotinin (6.5 kDa). The void volume of Superose 6 column was determined with Blue Dextran 2000 (2000 kDa).

### Negative-stain electron microscopy

*Rh*GmaS proteins (∼6 μg/ml) were applied to glow-discharged electron microscopy grids for 1 min and then stained with 2% (w/v) uranyl acetate twice for 30 s and 1 min, respectively. Negatively stained grids were imaged on a Talos L120C transmission electron microscope. (Thermo Fisher Scientific) operated at 120 kV. Images were recorded at ×73,000 magnification with a Ceta 16M camera (Thermo Fisher Scientific).

### Enzymatic activity assays

The enzymatic activity of GmaS was measured by determining the production of inorganic phosphate using a PiColorLock reagent kit (Expedeon, UK). The reaction system contained 200-mM glutamate, 25-mM MgCl_2_, 3-mM ATP, 1-mM MMA, and 100-mM Tris HCl (pH 8.0) and 2.5-nM *Rh*GmaS, 41-nM *R. pomeroyi* DSS-3 GmaS, or 2.8-nM *D*. *shibae* DFL12 GmaS. The reaction mixture was incubated at 60 °C for 10 min, and then, 20% (v/v) hydrochloric acid was added into the mixture to stop the reaction. The amount of inorganic phosphate in the mixture was determined by monitoring the absorbance at 635 nm. The optimal temperature for GmaS enzymatic activity was measured in a range from 20 to 80 °C at pH 8.0. The optimal pH was examined at 60 °C using Bis-Tris buffer for pH 6–7, Tris-HCl buffer for pH 7–9, and glycine-NaOH buffer for pH 9–10.5. The kinetic parameters of *Rh*GmaS (with and without the His-tag) for glutamate, ATP, and ammonia analogs were determined with the reaction system containing 14.3-nM *Rh*GmaS and different concentrations of substrates. All the measurements were carried out in 100-mM Tris-HCl buffer (pH 8.0) at 30 °C.

### Crystallization and data collection

Purified *Rh*GmaS protein was concentrated to ∼11 mg/ml in the buffer containing 10 mM Tris HCl (pH 8.0) and 100-mM NaCl. All of the crystals were obtained at 18 °C after 1–2 weeks using the sitting-drop vapor diffusion method (the ratio of protein to reservoir solution was 1:1). Apo-*Rh*GmaS crystals were obtained in the buffer containing 0.1-M MES (pH 6.5) and 1-M lithium sulfate. To obtain *Rh*GmaS–AMPPCP crystals, AMPPCP and MgCl_2_ were added to *Rh*GmaS with a final concentration of 2 mM, and the solution was mixed 1:1 with the crystallization buffer containing 0.1-M HEPES (pH 7.5), 0.2-M ammonium acetate, and 25% (v/v) isopropanol. To obtain the crystals of the *Rh*GmaS–AMPPNP–MetSox complex, *Rh*GmaS was cocrystallized with AMPPNP (2 mM), MetSox (2 mM), and MgCl_2_ (2 mM), and the crystals were obtained in the crystallization buffer containing 0.1 M bicine (pH 8.5), 10% (v/v) 2-propanol, and 30% (v/v) polyethylene glycol 1500. *Rh*GmaS–MetSox-P–ADP crystals were obtained under the same condition to the *Rh*GmaS–AMPPNP–MetSox crystals, with AMPPNP replaced by ATP. When *Rh*GmaS was cocrystallized with ATP (2 mM), glutamate (2 mM), and MgCl_2_ (2 mM), two structures of *Rh*GmaS in complex with ADP were obtained. The crystals of *Rh*GmaS–ADP-C1 were obtained in the crystallization buffer containing 1.1-M ammonium tartrate dibasic (pH 7.0), and the crystals of *Rh*GmaS–ADP-C2 were obtained in the crystallization buffer containing 0.1-M MES (pH 6.5) and 1-M lithium sulfate.

All X-ray diffraction data were collected on the BL18U1 beamline at the Shanghai Synchrotron Radiation Facility. The initial diffraction data sets were processed by the HKL3000 program with its default settings (35).

### Structure determination and refinement

The crystal structures of apo-*Rh*GmaS, *Rh*GmaS–AMPPCP, *Rh*GmaS–AMPPNP/MetSox, *Rh*GmaS–ADP–MetSox-P, *Rh*GmaS–ADP-C1, and *Rh*GmaS–ADP-C2 all belong to the I222 space group. The crystal structure of apo-*Rh*GmaS was determined by molecular replacement using the CCP4 program Phaser (36), with the structure of a GS enzyme from *B. subtilis* (PDB code: 4LNI) as the search model. All the other structures were determined by molecular replacement with the structure of apo-*Rh*GmaS as the search model. The refinements of these structures were performed using Coot (37) and *Phenix* (38). All the structure figures were processed using the program PyMOL (http://www.pymol.org/).

### Bioinformatics

Multiple sequence alignment was performed using CLC sequence viewer 6.5.3 (CLC Bio A/S). Phylogenetic tree was conducted using the MEGA, version 6.0 (39), with the neighbor-joining algorithms.

### CD spectroscopic assays

CD spectroscopic assays for *Rh*GmaS and all its mutants were carried out on a J-1500 Spectrometer (Jasco, Japan) at 25 °C. The concentration of the proteins was 8.5 μM in the buffer of 10-mM Tris HCl (pH 8.0) containing 100-mM NaCl. The spectra were collected from 250 to 200 nm at a scan speed of 500 nm min^−1^ with a band width of 1 nm.

## Data availability

The structures of apo-*Rh*GmaS, *Rh*GmaS–AMPPCP, *Rh*GmaS–AMPPNP–MetSox, *Rh*GmaS–ADP–MetSox-P, *Rh*GmaS–ADP-C1, and *Rh*GmaS–ADP-C2 have been deposited in PDB under accession codes 7CQL, 7CQN, 7CQQ, 7CQU, 7CQW, and 7CQX, respectively. All data are contained within the manuscript and the supporting information.

## Conflict of interest

The authors declare that they have no conflicts of interest with the contents of this article.
