# Supplementary material for: Crystal structures of γ-glutamylmethylamide synthetase provide insight into bacterial metabolism of oceanic monomethylamine
Source: J Biol Chem. 2020 Nov 21;296:100081. doi: 10.1074/jbc.RA120.015952 (PMC7948447; doi:10.1074/jbc.RA120.015952)
Supplement: Figures S1 and S2 [file mmc1.pdf]

Supporting information for:

Crystal structures of γ-glutamylmethylamide synthetase provide insight into bacterial metabolism of oceanic monomethylamine

Ning Wang, Xiu-Lan Chen, Chao Gao, Ming Peng, Peng Wang, Na Zhang, Fuchuan Li, Gui-Peng Yang, Qing-Tao Shen, Shengying Li, Yin Chen, Yu-Zhong Zhang, Chun-Yang Li*

*Corresponding author: Chun-Yang Li, lcy@ouc.edu.cn

This file includes:

Figs. S1 to S2

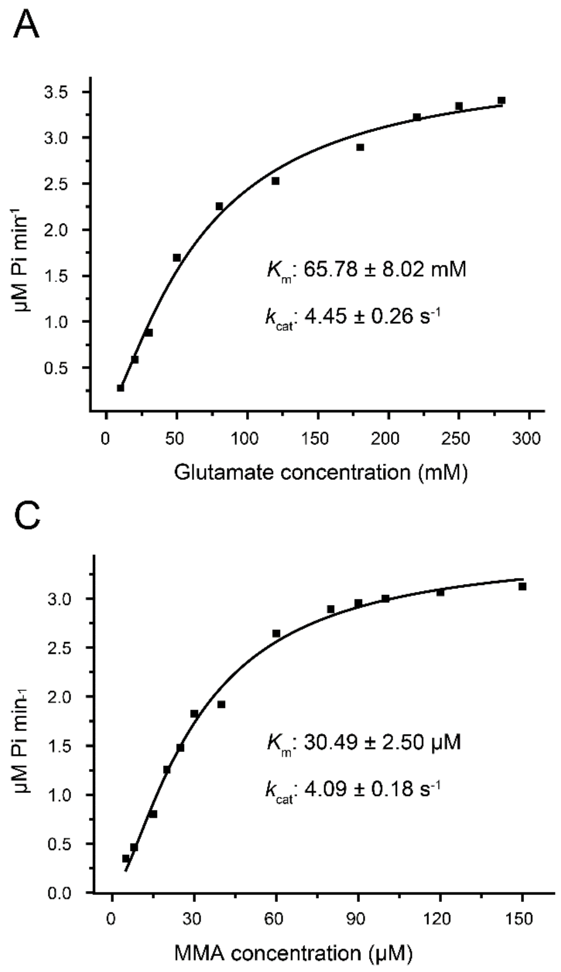

Fig. S1. Kinetic parameters of recombinant *Rh*GmaS without His-tag. A. Kinetic parameters of *Rh*GmaS for glutamate. B. Kinetic parameters of *Rh*GmaS for ATP. C. Kinetic parameters of *Rh*GmaS for MMA.

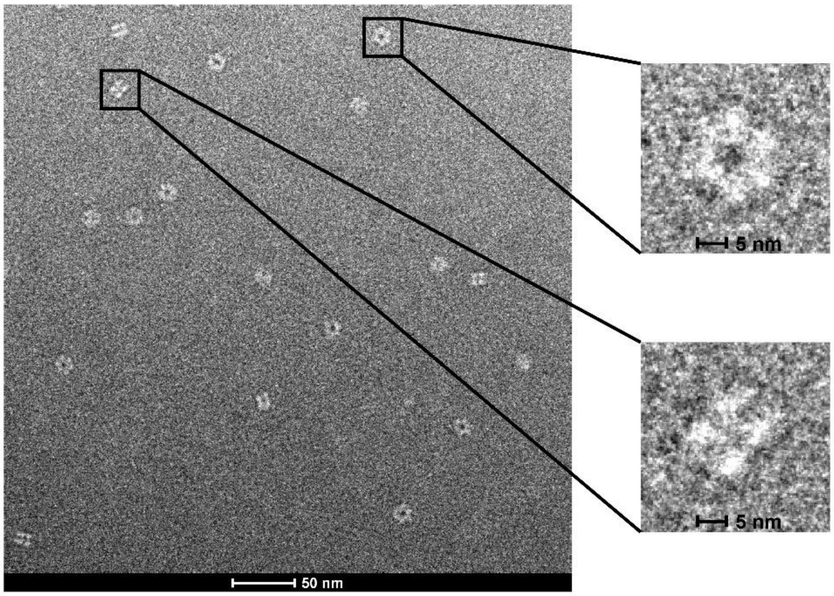

Fig. S2. The negative staining electron micrograph of *Rh*GmaS without his-tag.
